# A clarified position for *solanum lycopersicum *var. *cerasiforme *in the evolutionary history of tomatoes (solanaceae)

**DOI:** 10.1186/1471-2229-8-130

**Published:** 2008-12-20

**Authors:** Nicolas Ranc, Stéphane Muños, Sylvain Santoni, Mathilde Causse

**Affiliations:** 1 INRA, UR1052, Unité de Génétique et d'Amélioration des Fruits et Légumes, Montfavet 84 143, France; 2 INRA, UMR 1097 Diversité et Adaptation des Plantes Cultivées, Montpellier 34602, France

## Abstract

**Background:**

The natural phenotypic variability present in the germplasm of cultivated plants can be linked to molecular polymorphisms using association genetics. However it is necessary to consider the genetic structure of the germplasm used to avoid false association. The knowledge of genetic structure of plant populations can help in inferring plant evolutionary history. In this context, we genotyped 360 wild, feral and cultivated accessions with 20 simple sequence repeat markers and investigated the extent and structure of the genetic variation. The study focused on the red fruited tomato clade involved in the domestication of tomato and confirmed the admixture status of cherry tomatoes (*Solanum lycopersicum *var. *cerasiforme*). We used a nested sample strategy to set-up core collection maximizing the genetic diversity with a minimum of individuals.

**Results:**

Molecular diversity was considerably lower in *S. lycopersicum *i.e. the domesticated form. Model-based analysis showed that the 144 *S. lycopersicum *var. *cerasiforme *accessions were structured into two groups: one close to the domesticated group and one resulting from the admixture of the *S. lycopersicum *and *S. pimpinellifolium *genomes. SSR genotyping also indicates that domesticated and wild tomatoes have evolved as a species complex with intensive level of hybridization. We compiled genotypic and phenotypic data to identify sub-samples of 8, 24, 32 and 64 cherry tomato accessions that captured most of the genetic and morphological diversity present in the entire *S. lycopersicum *var. *cerasiforme *collection.

**Conclusion:**

The extent and structure of allelic variation is discussed in relation to historical events like domestication and modern selection. The potential use of the admixed group of *S. lycopersicum *var. *cerasiforme *for association genetics studies is also discussed. Nested core collections sampled to represent tomato diversity will be useful in diversity studies. Molecular and phenotypic variability of these core collections is defined. These collections are available for the scientific community and can be used as standardized panels for coordinating efforts on identifying novel interesting genes and on examining the domestication process in more detail.

## Background

Advances in molecular marker development and in genome mapping have resulted in high-density molecular-marker linkage maps in crops, and have provided tools for dissecting the genetic variation of complex traits. Map-based strategies were successfully used for the positional cloning of genes that underlie Quantitative Trait Loci (QTL) [1-3]. Despite the success of these strategies, gene discovery is still limited to those loci that have large effects upon quantitative variation [4].

Over the last few years, there has been renewed interest in the study of naturally occurring variation in crop genetic collections. Motivations for such studies are (i) to use natural allelic diversity for the evaluation of gene function, (ii) to find new genes or new alleles involved in specific aspects of plant physiology or development and (iii) to try to understand the molecular basis of adaptation to local environments [5]. Association genetics or linkage disequilibrium studies test for a statistical association between genotypes at a marker locus and the phenotypes in a set of unrelated individuals [6]. Polymorphisms of interest are detected in a large range of genetic backgrounds. The extent of linkage disequilibrium (LD), the non-random association of alleles at two or more loci, is a sample specific property and depends on the biological model studied. In contrast to the situation in multigenerational pedigrees, LD in natural populations is not broken artificially and we need to overcome this restriction.

The primary obstacle to successful association studies or linkage disequilibrium (LD) mapping is the nature of the genetic structure of populations [7]. The presence of subgroups with different allele frequencies, within the population studied, can lead to spurious associations. Domestication of most of modern crops occurred between 10,000 and 5,000 years ago and shaped the allelic frequencies distribution among plant populations. Knowledge about genetic structure can aid in inference of evolutionary history like domestication [8].

The large sample size to be analyzed constitutes another constraint in diversity studies, whereas studying a subset might be more efficient if this sample spans the full range of variation [9]. The first challenge in molecular diversity analysis is thus to sample core collections that better fit the range of morphological and genetic variations found in the global collection. For example, Single Nucleotide Polymorphism (SNP) candidate markers, discovered in a small number of accessions, can be easily genotyped on a larger sample for diversity analysis and association mapping. Several methods have been proposed for constructing core-collections. Some of these take advantage of molecular markers [10] and seem to perform better when used for sampling autogamous plants [11]. The genetic structure of a core collection has to be checked to avoid spurious correlation between molecular polymorphisms and traits in association studies.

Tomato (*Solanum lycopersicum*, formerly *Lycopersicon esculentum*) emerged as a model species for the study of fleshy fruited plants because of the extent of genetic and genomic resources available [12,13]. The large range of phenotypic variation and large collections of genetic resources available for crops are prerequisites for using an association strategy. The cultivated tomato is highly autogamous and shows a large range of morphological diversity but low genetic diversity compared to other *Solanum *relatives [14]. This can be explained by successive bottlenecks: (i) domestication associated with isolation of the crop from the Andes (centre of diversity) to Central America, (ii) transfer of few cultivars to the Mediterranean basin by conquistadors in the 16^th ^century and (iii) modern breeding [15]. Cherry tomato, i.e. *S. lycopersicum *var. *cerasiforme *(*S. l. cerasiforme*), is the expected ancestor of the domesticated form. In its native Andean region, wild and feral forms can be found and *S. l. cerasiforme *is also described as highly invasive [16]. Cherry tomato accessions are also found as landraces from temperate to subartic regions. In Coastal Ecuador and Peru, *S. pimpinellifolium*, genetically close to *S. lycopersicum *and strictly wild, is found growing in sympatry with tomato landraces and cherry tomato (and also with *S. peruvianum *and *S. hirsutum*, two green-fruited species). Wild and feral *S. l. cerasiforme *(i.e. cherry type) exhibit two allozyme-diversity patterns: one similar to the allozyme-diversity pattern exhibited by cultivated tomato and another one similar to the wild *S. pimpinellifolium *allozyme-diversity pattern [17]. Based on isozymes, *S. l. cerasiforme *accessions also show an outcrossing rate comparable to the rate of outbred species [18]. Rick and Holle (1990) suggest that tomato should have undergone natural introgressions from wild and feral accessions. Moreover, Nesbitt and Tanksley [19] demonstrated that, around the *fw2.2 *locus, the *S. l. cerasiforme *genome is a mosaic between *S. lycopersicum *and *S. pimpinellifolium *genomes due to frequent hybridizations between the two species. This is evidence of frequent hybridizations in this autogamous complex of species. The admixture hypothesis of *S. l. cerasiforme *has never been tested on the whole genome and would be further evidence of a natural high rate of hybridization. Moreover, *S. l. cerasiforme *and *S. pimpinellifolium *are involved in the domestication of tomato but the process remains to be clarified.

Molecular markers like simple sequence repeat (SSR) markers have often been used to clarify genetic structure in plants [20-25]. In tomato several studies used SSR markers but focused only on wild relatives [26,27] or on elite germplasm [28,29]. No study used a broad sample of cultivated, landraces, and wild accessions. The goal of the present study is to clarify the domestication process of tomato and to confirm the admixture status of *S. l. cerasiforme*. To achieve this goal we analyzed the genetic structure of a genetic resource collection, that includes predominantly *S. l. cerasiforme *accessions, and we compared this to the genetic structure of *S. lycopersicum *and *S. pimpinellifolium*. We assessed the amount of genetic diversity in the collection and sampled nested core collections of wild and cultivated tomato that will be used in future diversity studies. For this purpose we used a set of 20 SSR markers dispersed over the genome to survey the genetic diversity present in a sample of 360 accessions.

## Results

### Microsatellite diversity

The Microsatellite markers used (table 1) revealed different diversity patterns in the total collection including green fruited species, *S. cheesmaniae *(N = 20) and red-fruited accessions (N = 340) (table 2). SSR markers revealed 2 to 26 different alleles and an average of 12.45 alleles per locus. This mean dropped to 3 alleles per locus when rare alleles (i.e. with a frequency lower than 0.05) were removed. In the red-fruited tomatoes group, the average allele number per locus was N_A _= 7.7 but was equivalent to the total collection when removing rare alleles (N_A _= 3.3). The average expected heterozygosity over all loci was 0.496 with large variation among loci (SD = 0.225). Rare heterozygous genotypes were found for all loci in the total collection (H_O _> 0) but were distributed across individuals.

**Table 1:** Characteristics of microsatellite loci

**Locus** **name**	**Motif**	**Linkage** **group^a^**	**Map** **position^a ^** **(cM)**	**Primer sequences (5'- 3')**
SSR599	[TCATTA]_2_[TCA]_6_	9	103.00	GGATTTCTCATGGAGAATCAGTC
				TCCCTTGATCTTGATGATGTTG

SSR111	[TC]_6_[TCTG]_6_	3	73.90	TTCTTCCCTTCCATCAGTTCT
				TTTGCTGCTATACTGCTGACA

SSR14	[ATA]_9_	3	162.50	TCTGCATCTGGTGAAGCAAG
				CTGGATTGCCTGGTTGATTT

SSR248	[TA]_21_	10	35.00	GCATTCGCTGTAGCTCGTTT
				GGGAGCTTCATCATAGTAACG

SSR52	[AAC]_9_	7	3.00	TGATGGCAGCATCGTAGAAG
				GGTGCGAAGGGATTTACAGA

SSR150	[CTT]_7_	1	115.50	ATGCCTCGCTACCTCCTCTT
				AATCGTTCGTTCACAAACCC

SSR117	[TC]_11_	1	138.00	AATTCACCTTTCTTCCGTCG
				GCCCTCGAATCTGGTAGCTT

SSR66	[ATA]_8_	2	25.00	TGCAACAACTGGATAGGTCG
				TGGATGAAACGGATGTTGAA

SSR136	[CAG]_7_	11	11.00	GAAACCGCCTCTTTCACTTG
				CAGCAATGATTCCAGCGATA

SSR578	[AAC]_6_[ATC]_5_	6	44.00	ATTCCCAGCACAACCAGACT
				GTTGGTGGATGAAATTTGTG

SSR47	[AT]_14_	6	6.50	TCCTCAAGAAATGAAGCTCTGA
				CCTTGGAGATAACAACCACAA

SSR594	[TCT]_8_	8	55.00	TTCGTTGAAGAAGATGATGGTC
				CAAAGAGAACAAGCATCCAAGA

SSR22	[AT]_11_	3	99.00	GATCGGCAGTAGGTGCTCTC
				CAAGAAACACCCATATCCGC

SSR327	[AAT]_7_	8	22.50	TCAGGATCAGGAGCAGGAGT
				TGGACTTGTTCCATGAACCC

SSR593	[TAC]_7_	4	15.00	TGGCATGAACAACAACCAAT
				AGGAAGTTGCATTAGGCCAT

SSR26	[CGG]_7_	2	77.50	CGCCTATCGATACCACCACT
				ATTGATCCGTTTGGTTCTGC

SSR45	[AAT]_14_	7	80.00	TGTATCCTGGTGGACCAATG
				TCCAAGTATCAGGCACACCA

SSR20	[GAA]_8_	12	37.00	GAGGACGACAACAACAACGA
				GACATGCCACTTAGATCCACAA

SSR70	[AT]_20_	9	42.00	TTTAGGGTGTCTGTGGGTCC
				GGAGTGCGCAGAGGATAGAG

SSR188	[AT]_11_	4	135.50	TGCAGTGAGTCTCGATTTGC
				GGTCTCATTGCAGATAGGGC

^a ^Linkage group and map position are based on the tomato EXPEN 2000 map .

**Table 2:** Microsatellite diversity detected in the total collection and in the red fruited subgroup

**Locus name**	**N_A _^a^**			**N_A, P _^b^**			**H_E _^c^**			**H_O _^d^**	
	
	**total**	**red-fruited**		**total**	**red-fruited**		**total**	**red-fruited**		**total**	**red-fruited**
SSR599	6	4		1	1		0.117	0.023		0.011	0.002

SSR111	14	8		4	5		0.654	0.615		0.033	0.029

SSR14	11	9		4	4		0.621	0.603		0.036	0.035

SSR248	25	18		7	8		0.899	0.888		0.067	0.050

SSR52	5	2		1	1		0.070	0.012		0.003	0.000

SSR150	10	6		2	2		0.294	0.220		0.022	0.021

SSR117	13	6		3	3		0.533	0.478		0.050	0.038

SSR66	8	4		3	3		0.421	0.363		0.017	0.024

SSR136	8	6		4	3		0.457	0.396		0.036	0.029

SSR578	6	2		2	2		0.372	0.309		0.008	0.009

SSR47	26	25		3	3		0.725	0.710		0.046	0.048

SSR594	13	5		2	3		0.517	0.463		0.042	0.032

SSR22	17	9		2	3		0.580	0.532		0.061	0.047

SSR327	14	5		2	2		0.275	0.219		0.039	0.021

SSR593	9	6		2	5		0.574	0.537		0.047	0.038

SSR26	2	2		2	2		0.452	0.425		0.017	0.018

SSR45	20	14		5	5		0.795	0.776		0.081	0.062

SSR20	4	3		3	3		0.334	0.281		0.031	0.021

SSR70	21	17		5	5		0.848	0.832		0.069	0.065

SSR188	17	3		3	3		0.386	0.324		0.025	0.015

Mean (SD)	12.45	7.7		3	3.3		0.496 (0.225)	0.4503 (0.2442)		0.037 (0.022)	0.0303 (0.0178)

^a ^number of allele per locus (^b ^number of allele with frequency higher than 5%), ^c ^expected heterozygosity, ^d ^observed heterozygosity

A much higher genetic diversity was found in wild *S. pimpinellifolium *(H_E _= 0.58) than in the cultivated *S. lycopersicum *(H_E _= 0.25) (table 3). The observed heterozygosity was also higher for *S. pimpinellifolium *(H_O _= 0.0591) than for *S. lycopersicon *(H_O _= 0.0098). The reason for these heterozygosity patterns could be the difference in the reproductive regime between *S. pimpinellifolium *accessions and *S. lycopersicum*. The *S. l. cerasiforme *exhibited an intermediate pattern of diversity.

**Table 3:** Pattern of genetic diversity inferred from simple sequence repeat markers among tomato species.

**sample**	**number of** **individual**	**Total ** **number** **of alleles**	**specific** **allele** **number^a^**	**H_E _^b^**	**H_O _^c^**
*S. lycopersicum*	130	88	6	0.2479	0.0098
*S. lycopersicum *var.*cerasiforme*	144	99	6	0.3816	0.0370
*S. pimpinellifolium*	66	130	13	0.5781	0.0591
*total red-fruited sample*	340	154	-	0.4503	0.0300

^a ^specific alleles are identified when they are found only in one sample. ^b ^expected heterozygosity, ^c ^observed heterozygosity

### Genetic structure of the sample

The genetic structure in the red-fruited accession sample was analyzed with the model-based clustering algorithm implemented in the Structure2.0 software (see Methods section for details). To avoid redundancy in the collection, we kept only one individual when several accessions were identified with the same SSR fingerprint at all loci. Hence, 23 individuals (18 *S. lycopersicum *and 5 *S. pimpinellifolium*) were removed. Thus, we detected the genetic structure of a sample of 318 accessions. Because *S. l. cerasiforme *genome was described as a mosaic between *S. lycopersicum *and *S. pimpinellifolium *genomes, all the red-fruited accessions were used as a broad sample. *S. cheesmaniae *and *S. galapagense *accessions have not taken part in the domestication process of tomato and were not included in this analysis.

**Figure 1:**
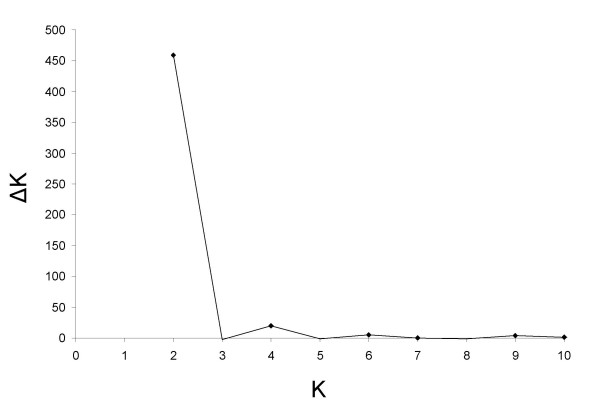
**Determination of Kopt following the method of Evanno et al. (2005)**. The rate of change of the posterior probability of the data given the number of clusters is plotted against K, the number of clusters. ΔK was calculated as |L"(K)|/s[Pr(x|k)] (see Materials and Methods). The first peak (K = 2) corresponds to the optimum number of clusters. The secondary peak (K = 4) indicates a sublevel clustering.

The Evanno et al. (2005) correction of the Structure2.0 outputs was used (Figure 1). The first peak of ΔK, for K = 2, corresponded to the presence of two main clusters and a potential sublevel of clustering was suggested by the secondary peak of ΔK, for K = 4. The classification of accessions into clusters by the model-based method was used to study the sublevel clustering of the red-fruited tomato sample. For all K_opt_, memberships were consistent between all runs.

For K_opt _= 2, clustering divided the total sample into two groups. Group 1 consisted of the main part of *S. pimpinellifolium *(Table 4) with 20 accessions from *S. l. cerasiforme *whereas group 2 consisted of the main part of S. *lycopersicum *and of the *S. l. cerasiforme *samples. Group 1 represented the 'wild' part of the sample whereas group 2 represented the 'domesticated' part of the sample. This classification accounted for 35% (p < 0.000001) of the total genetic variance; individuals within group accounted for 51% (p < 0.000001) of the total variance and the variance within individuals explained five percent (p < 0.000001) of the total variance. When individuals were assigned with a minimal membership of 70% into a corresponding cluster, twenty three percent (i.e. 35 individuals) of the *S. l. cerasiforme *accessions was in admixture between 'wild' and 'domesticated' groups.

**Table 4:** Species classification among clusters described by Structure2.0 based on maximal individual-membership for each cluster.

**species**	**K_opt _= 2**			**K_opt _= 4**			
	
	Pop1	Pop2		popA	PopB	PopC	popD
*S. lycopersicum*	1	112		1	0	23	89

*S. lycopersicum *var. *cerasiforme*	20	124		13	10	78	43

*S. pimpinellifolium*	58	3		21	35	3	2

Number of pairs of loci in LD (# of comparisons)^a^	158(189)	87(189)		62(152)	143(189)	74(135)	58(189)

^a ^Pairs of markers were considered in significant LD using the threshold p-value < 0.001.

For K_opt _= 4, the group 1 divided into subgroups A and B and the group 2 divided into subgroups C and D. When individuals with a membership lower than 70% were not taken into account, the hierarchical AMOVA indicated that 37% (p < 0.000001) of the variance was due to variation among groups, 13% (p < 0.000001) of the variance was due to variation among subgroup within groups and 45% (p < 0.000001) of the variance was due to variation among individuals within subgroup (only five percent (p < 0.000001) was due to variation within individual). Pairwise estimates of F_ST _indicated a high degree of differentiation between the four clusters with values ranging from 0.21 between clusters C and D to 0.64 between clusters A and D (Table 5).

**Table 5:** Subgroup pairwise F_ST _for K_opt _= 4

	popA	popB	popC
popA	0.00000		
popB	0.23358	0.00000	
popC	0.57111	0.39977	0.00000
popD	0.64958	0.48797	0.21444

Individuals were clustered corresponding to their maximal membership. All comparisons were significant (p < 0.001)

The cluster A consisted of moderate to large fruited individuals with a large part of *S. lycopersicum *accessions, whereas cluster B consisted of small fruited accessions with the cherry type accessions representing the main part of this subgroup. The 'wild' group was divided into the cluster A and B; both consisted of *S. l. cerasiforme *and *S. pimpinellifolium *accessions. When individuals were assigned with a minimal membership of 70% into a corresponding cluster, individuals were found in admixture between intra-specific groups but most admixture accessions were inter-specific admixes (Table 6).

**Table 6:** Individual clustering, allelic diversity and proportion of loci in linkage disequilibrium in the four clusters inferred using Structure2.0 (n = 318)

	**Group 1: 'wild'**				**Group 2: 'domesticated'**			**'wild/domesticated'** **Admixed**
	
**Number of individuals** **in each cluster**	**Subgroup** **A**	**Subgroup** **B**	**AB** **Admixed**		**Subgroup** **C**	**Subgroup** **D**	**CD** **Admixed**	
*S. lycopersicum*^a^	1	0	0		16	81	12	3

*cerasiforme*^a^	3	6	1		59	19	22	34

*S. pimpinellifolium*^a^	13	30	7		1	0	0	10

total	17	36	8		76	100	34	47

**N_A _^b^**	2.9	6.2	2.8		4.15	3.4	3.3	4.7

**H_E _^c^**	0.3275	0.5960	0.3852		0.2816	0.2245	0.2772	0.4595

**LD (number of comparison)**	23/135	135/190	16/90		36/119	25/152	80/119	83/152

^a ^Individuals were classified in a cluster if their membership for this cluster was higher than 70%. ^a ^number of individual in each cluster. ^b ^allele number. ^c ^Expected heterozygosity

**Figure 2:**
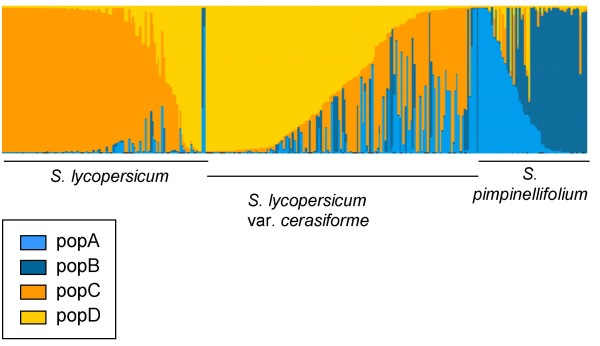
**Classification of individuals using *Structure2.0 *according to the previous classification into species**. The distribution of the individuals to different clusters by the model-based method is indicated by the color code in the legend box.

Groups 1 and 2 were considered as main samples and analyzed separately using the same hypothesis. The optimum number of sublevel populations within the groups 1 and 2 was two, which is consistent with the K_opt _of 4 for the whole sample. Classification of individuals in each cluster was consistent with results based on Structure2.0 outputs of the total sample. For K_opt _= 4, there were differences between individual's memberships and species classification (Figure 2). Some individuals were misclassified.

We also analyzed the genetic structure of each species separately (see Additional file 1: Determination of Kopt for each species) and the memberships of individuals was consistent with clustering found in the whole red-fruited tomato sample. Individuals previously found in admixture clustered in independent groups.

The pattern of genetic diversity within the subdivision was analyzed (Table 6). The two 'wild' clusters presented the highest H_E _but subgroup A had a low value of H_E _compared to subgroup B. The numbers of statistical pairwise comparisons for non random association of alleles (Table 6) are homogeneous among subgroup A, C and D but much higher for subgroup B and for the 'wild' and 'domesticated' admixed part of *S. l. cerasiforme*. The clustering allowed linkage disequilibrium to decrease in each subgroup compared to the whole sample.

**Figure 3:**
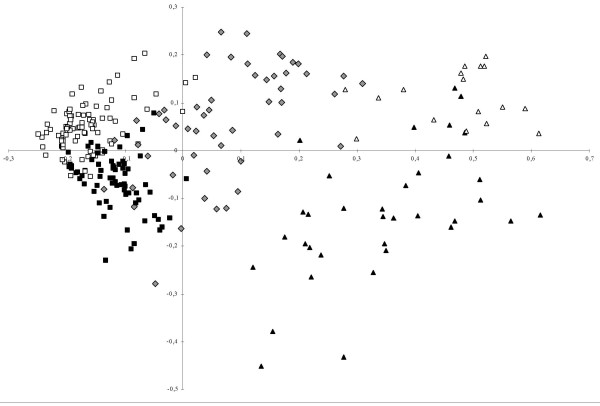
**Principal Coordinate Analysis of the *Eulycopersicon *sample with Structure2.0 clustering information**. The 'red-fruited' sample did not contain *S. cheesmaniae *accessions. The subdivision of the collection assuming Kopt = 2 separates group 1 (triangle) and group 2, (square) accessions. When assuming Kopt = 4, large fruited accessions: subgroup D (black square) and small-size fruit accessions: subgroup C (white square) are divided. For wild accessions, subgroup A (white triangle) and subgroup D (black triangle) were divided. 'Wild'/'domesticated' admixed accessions are represented by grey diamonds. Intra-specific admixed accessions are not identified. Inertia values are 22.09% and 4.84% for factorial coordinates axes 1 and 2, respectively.

The first axis of Principal Coordinate Analysis of the red-fruited tomatoes separated 'wild' *S. pimpinellifolium *from 'domesticated' *S. lycopersicum *(Figure 3). The second axis separated subgroups A and B on one hand and subgroups C and D on the other hand. The *S. l. cerasiforme *accessions were divided among subgroups B and the admixed cluster. The interspecific admixed group showed a *continuum *between 'wild' and 'domesticated' clusters.

### Sampling of the Core collection

**Figure 4:**
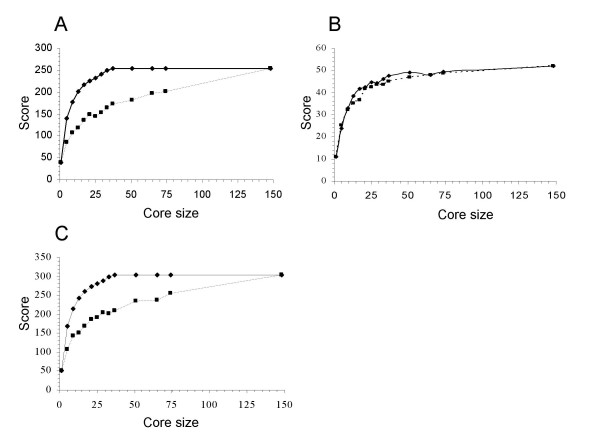
**Comparison of efficiency of random and maximization (M) sampling strategy in *S. l. cerasiforme *sample (n = 143 accessions)**. Score, which represents allelic richness, is plotted against size of core collection. The efficiency of the M strategy is represented by a straight line and the random strategy is represented by a dashed line. A. Core collections were sampled with alleles from 20 SSR loci and were cross validated by the same alleles. B. Core collections were sampled with alleles from 20 SSR loci and were cross validated by alleles from twelve phenotypic data split in 5 classes. C. Core collections were sampled with alleles from 20 SSR markers and twelve phenotypic data and were cross validated by the same alleles.

Core collections of *S. l. cerasiforme *accessions were built using the Maximization or M strategy algorithm implemented in MStrat software v.4.1. Analyses were first performed on all cherry tomato accessions only (144 accessions). Before sampling the core collections, the whole sample was analyzed to compare two sampling strategies. We also determined the size of the smallest subset that captured all molecular and phenotypic alleles present in the whole sample. Both molecular and phenotypic data were used for these analyses. The phenotypic quantitative variables were split into 5 classes of equal dimension (see Methods). Random and M sampling strategies were compared. SSR allelic richness (number of alleles captured if sampling a core collection of n individuals) was calculated for each core collection size. The 20 SSR alleles were used both as markers, to implement the M and random strategy, and target variables, to compare these two strategies (Figure 4a). The difference between the random and M curves indicated that the M strategy performed better in sampling a core collection for the *S. l. cerasiforme *sample. The optimal size for the core collection, obtained at the plateau of the M curve, was reached for 37 *S. l. cerasiforme *accessions.

The phenotypic diversity captured when sampling only with SSR alleles is shown in figure 4b. The plateau of the M curve was reached for 51 individuals and a weak difference in performance between the two strategies was observed.

When both molecular and phenotypic data were used as marker variables (i.e. to sample the core collection), the M strategy showed higher performance in sampling procedure than a random strategy and gave an optimal size of 51 individuals (figure 4c). Finally, core collections were sampled using both molecular and morphological data. To define the final core collection, accessions were classified by the number of times they were sampled in the fifteen replicates and the most frequently sampled accessions were chosen.

Four nested core collections composed of 8, 24, 32 and 64 *S. l. cerasiforme *accessions were sampled (see Additional file 2: Cerasiforme and mixed core collections). Fourty to 98% of SSR alleles were captured when accession's number increased from 8 to 64 (table 7). The number of phenotypic classes captured, increased from 18 (60% of the classes from the *S. l. cerasiforme *sample) to 27 (90% of the classes from the *S. l. cerasiforme *sample) when accession's number increased from 8 to 64. The 64 accession sample did not show any genetic structure when it was analyzed with the model-based method.

**Table 7:** Phenotypic and molecular representativeness of the four *cerasiforme *core collections.

	SSR allele		Number of classes for phenotypic quantitative trait^a^						
Size of core collection	number	%	Active variables					Target variables^b^		Total
				
			Fruit weight	Fruit locule number	Firmness	Color (a*)^c^		SSC^c^	titratable acidity^c^	

8	52	40	3	2	4	2		4	3	18

24	105	76.9	4	3	5	4		4	3	23

32	119	91.5	4	3	5	5		4	5	26

64	128	98.5	4	3	5	5		5	5	27

total	130	100	5	5	5	5		5	5	30

^a ^Each quantitative variable was split into 5 classes of equal dimension (see Materials and Methods) when looking for the whole *cerasiforme *sample and number of classes. ^b ^Soluble Solid Content (SSC) and titratable acidity were only used to analyse core collections representativeness and were not used as active variables for the sampling of these core collections. ^c ^a* describes how red/green a color is.

**Figure 5:**
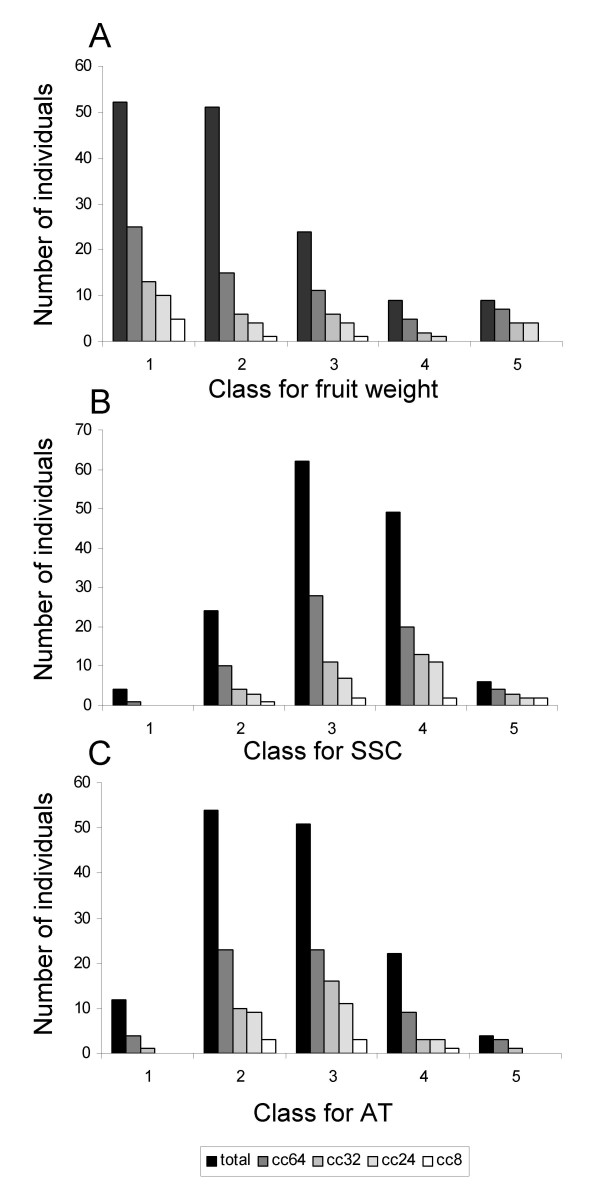
**Core collection representativeness for fruit weight, Soluble Solid Content and Titratable Acidity**. Classes are those used for core collections (cc) design.

For fruit weight (FW), soluble solid content (SSC) and titratable acidity (TA), the core collection of 64 accessions best represented the phenotypic variability of the global sample even though extreme phenotypes were not represented (Figure 5). The sample consisting of 32 accessions seemed to be the best compromise because of its small number of accessions and its representativeness.

To complete representativeness of these core collections, ten accessions from *S. lycopersicum*, two accessions from *S. pimpinellifolium *and four wild related accessions (*S. chesmaniae, S. habrochaites, S. pennellii and S. chmielewskii*) were added to each of the core collections to constitute mixed interspecific core collections. The core collection of 64 accessions was also completed with seven other accessions from *S. lycopersicum *and eight accessions from *S. pimpinellifolium *also sampled separately using 20 SSR alleles and 12 morphological traits with the M strategy.

## Discussion

Previous studies on the genetic structure of tomato collections focused on cultivated accessions [29,30] or on the relationship between cultivated and wild relatives [26,27] but did not use a broad sample of wild and cultivated tomatoes with *S. l. cerasiforme *as the main sample. SSR markers have already been shown to be useful for genetic analysis in studies focusing on inferring interspecific relationships or confirming SSR reliability for genetic mapping [26,28,31-34].

Differences were observed among SSR markers. For example, a higher number of alleles was identified in the two-base motif markers compared to other three-base or complex motif markers (P-value = 0.039). A significant difference was observed between the number of alleles with AT-rich motifs and non AT-rich motif markers (P-value = 0.032). Two base AT-rich motif markers also displayed higher expected heterozygosity. This kind of SSR marker might be useful for inferring fine relationships between close accessions. Because of the higher mutation rate in the AT-rich motif markers, some misevaluation might occur because of homoplasy (i.e. alleles identical in terms of state but not by descent) for distant individuals [35]. SSR markers with lower mutation rates with three-base or complex motifs are more reliable markers for inferring interspecific relationships.

SSR markers had between two and 26 different alleles in the total collection (including eighteen wild green-fruited accessions, one *S. galapagense *and one *S. cheesmaniae *accessions) and the allele number decreased between one and five alleles when looking in the red-fruited tomato sample and only for allelic frequency higher than 5%. The pattern of genetic diversity inferred from SSR alleles also showed an important decrease in diversity (i.e. expected heterozygosity) when comparing *S. pimpinellifolium *and *S. lycopersicum *accessions. Furthermore, the observed heterozygosity is lower than expected for all species due to the reproductive regime of red-fruited accessions, but also to the way genetic resources were maintained. The red-fruited accessions are mainly autogamous (except a few highly allogamous *S. pimpinellifolium *accessions) and the green fruited accessions are mainly self-incompatible (except *S. chmielewskii *and *S. neorickii *which are self-compatible) [27]. The decrease of allele number and diversity in red fruited accessions is probably due to the restriction of allogamy. The drop in diversity between the wild and domesticated species has been previously described [14,16,17] and was explained by successive bottlenecks starting from domestication and continuing with modern breeding of *S. lycopersicum*. This species presents a high selfing rate which hampers restoration of genetic diversity lost during domestication. *S. pimpinellifolium *showed higher diversity because of its wild status (weak anthropic restriction in the effective population size compared to domesticated species) and because it benefited from intercrossing. In fact, partial allogamous populations of *S. pimpinellifolium *were described in Northwestern Peru. While they migrated away from that territory, selection has favored self pollination [36]. The higher rate of observed heterozygosity shown by *S. pimpinnellifolium *is thus a residue of intercrossing from allogamous accessions.

All red-fruited plants used are progenies from self-compatible accessions where seeds are produced through self-pollination. Because of the inbred nature of most accessions, only one plant was used for genotyping. The amount of diversity in this sample is thus underestimated. There is a bias when analyzing observed heterozygosity on artificially self-pollinated accessions but the residue of intercrossing observed testifies to ancestral intercrossing. The estimation of observed heterozygosity should be done on the initial population (from prospecting) to assess the intercrossing rate of these populations. However, the homozygosity of accessions will help in dissecting the genetic bases of agronomical traits using diversity studies.

*S. l. cerasiforme *showed an intermediate amount of genetic diversity between *S. lycopersicum *and *S. pimpinellifolium*. This particular position has already been described using allozymic variation [17] and both patterns of genetic variation close to *S. pimpinellifolium *and S. *lycopersicum *were encompassed. Cherry type tomatoes, found in coastal Peru or Ecuador and which were described as feral, wild, or used as cultivated landraces, may have played an important role in the evolution of domesticated tomato [37]. This variety characterized by morphological traits like fruit size and seed weight spans a genetic *continuum *between 'wild' and 'domesticated' forms of the crop. Current results suggest that this group of *S. l. cerasiforme *evolved through hybridization between *S. lycopersicum *and *S. pimpinellifolium*. The wild and feral parts of *S. l. cerasiforme *accessions, which have been described as highly invasive, adapted rapidly thanks to the increase in genetic variance, new gene interactions, masking or unloading of deleterious recessive alleles, or the transfer of favourable genes [38].

Genetic structure was highlighted by the model-based method developed by Pritchard et al., (2000) for human genetics. This method performed better than clustering methods based on pairwise genetic distance because only a modest number of loci was used [6]. The higher level of genetic structure allowed most of the *S. lycopersicum *and a part of *S. l. cerasiforme *accessions to be assigned to a 'domesticated' group and most of the *S. pimpinellifolium *to the 'wild' group. The other part of the cherry tomato sample was classified in an admixture position, which is consistent with the distance-based method. The subdivision of the 'domesticated' group in large and small fruit size accessions is consistent with the results of van Berloo et al. (2008) with AFLP markers. These authors found higher differences between cherry versus beef and round tomatoes than between round and beef tomatoes themselves. Homozygosity creates departure from Hardy-Weinberg equilibrium which is one of the hypotheses to apply the model-based method. This limitation was overcome using haploid genotypes. Simulations showed that dominant markers can give results as accurate as codominant markers [39]. We can thus validate our clustering though genotypes were coded in haploid setting. However, caution must be exerted when interpreting biological significance of the clustering because results are sensitive to the type of genetic marker used, the number of loci scored, the number of population sampled and the number of individual typed in each sample.

No relationship between the geographical origin and genetic structure was found within the wild group. Geographic distributions of genetic variability were highlighted for *S. pimpinellifolium *across coastal Peru and Ecuador, using isozyme markers [36]. Regional distribution of isozyme allelic variants and morphological traits for *S. l. cerasiforme *was also described [17,18]. This could be explained by differences in property for markers used. Allozyme markers and morphological traits may be under selective constraint in natural populations in contrast with SSR markers which are usually described following the neutrality hypothesis. Moreover, the results cited above, were obtained for offspring directly collected from natural populations. We employed a different approach using highly inbred plants: diversity patterns were compared among clusters and not among natural populations. The SSR markers presented in this study should be genotyped for natural populations of *S. pimpinellifolium *or *S. l. cerasiforme *to elucidate the correlation of geographical and genetic structures.

The lower amount of diversity and the highest number of alleles in LD in the subgroup A could be explained by reproductive isolation with a high frequency of short-style flowers in the original population (data not shown). This trait is characteristic of strictly autogamous tomato accessions [16,40]. This morphological change, that favors selfing over outcrossing, could also explain the genetic structure [41]. The higher genetic diversity of subgroup C in the 'domesticated' groups could be due to a more ancient and less drastic genetic bottleneck caused by domestication. The drop in genetic diversity in subgroup D is likely due to modern selection which focused on yield and fruit size. The interspecific admixed cluster presented high value of diversity index which is inconsistent with highly autogamous and domesticated forms but confirmed the hypothesis of frequent recombination between cultivated *S. lycopersicum *and wild *S. pimpinellifolium*. These results suggest a two-step selection for fruit size during domestication of tomato from *S. pimpinellifolium *to *S. lycopersicum*. A first step may have allowed selection of cherry type with moderate fruit size probably with fixation of autogamy. The human migration may have resulted in transfer of cultivated tomato from the Andes to Central America with selection for larger fruit size. In Mexico, tomato reached a fairly advanced stage of domestication before being taken to the Old World by conquistador [15,42]. The role of the 'admixed' part of *S. l. cerasiforme*, in tomato domestication can't be established because hybrid pattern could be due to ancient or recent outcrossing events.

The admixed *S. l. cerasiforme *cluster is of particular interest for mapping complex traits. This subsample could be used in an admixture mapping strategy that falls between linkage analysis and association mapping, and is a good approach for initial genome scan [43]. The extent of difference in allele frequency between the ancestral populations is crucial in detecting strong associations between phenotypes and molecular polymorphisms. This difference in allele frequency was obvious in 'wild' and 'domesticated' tomato groups as it represented the main genetic structure level highlighted with the model-based method. In humans, admixture mapping has already been performed to map two loci responsible for hypertension [44]. This method will be assessed in future studies for identifying new QTLs or candidate genes linked to fruit quality traits.

The number of pairwise markers at linkage disequilibrium (LD) decreased in the different groups compared to the total red-fruited accessions. Strong LD between distant or independent markers arises as a consequence of genetic linkage, of the rate of recombination, drift or non-random mating, and as a consequence of population structure. Information on genetic structure of the collection and the membership information for all individuals will be useful in future association mapping to avoid spurious associations due to strong LD over the genome [6].

However, more markers are needed to efficiently tag the genome and better unravel the genetic structure of the cultivated *S. lycopersicum *and *S. l. cerasiforme*. Furthermore, more markers will also be of great interest for estimating individual's kinships. New statistical methods for association studies use both genetic structure information and kinship estimation [45].

This study provided a set of nested core collections for *S. l. cerasiforme *accessions which was completed by selected accessions of *S. lycopersicum*, *S. pimpinellifolium *and wild relatives representing parents of different mapping populations. We focused on *S. l. cerasiforme *because of (i) its close relationship with *S. lycopersicum*, (ii) its genetic diversity which is higher than that of *S. lycopersicum *and (iii) its high range of variation in fruit quality traits. Because of differences in genetic and morphological diversity patterns in 'wild' versus 'domesticated' forms of the tomato *continuum*, core collections were sampled using both phenotypic and molecular diversity. For sampling core collections, the gain when scoring with the Maximization strategy was higher than with the Random strategy. This is not surprising given the high level of selfing in *S. l. cerasiforme *and the pattern of genetic structure uncovered in our sample, both factors that favor the marker assisted sampling strategies over pure random strategies [10,11]. Moreover 20 SSR markers were not sufficient to differentiate all accessions based on their genotype. Markers with higher mutation rates will be more accurate in differentiating individuals based on fingerprinting but will decrease the accuracy of sampling core collections with the M strategy.

Moreover, the M strategy sampled molecular diversity but also morphological diversity even for traits that were not used as markers for sampling the collection.

The four core collections proposed will have different goals. The 24 mixed core collection (including cultivated and wild mapping population parents) will be useful for detecting SNPs by sequencing. SNP markers will then be genotyped on the whole tomato collection for association studies or on mapping populations for QTL localization. Sampling this collection was a preliminary step for future studies on exploring the natural diversity of tomato that will unfold as the tomato genome sequence becomes available [13]. For example, Simon et al. [46] crossed *Arabidopsis thaliana *reference genotypes (i.e. whole genome sequenced genotypes) with several accessions from a previously defined core collection [47]. The authors built 15 Recombinant Inbred Line family and this new RIL set offered improved accuracy for QTL localization than previous RIL families.

The 64 *S. l. cerasiforme *core collection will be useful for direct association studies. This core collection maximizes the power of associations between phenotypes and allele frequencies. The core collection was test with the model based methods and showed no genetic structure. A broad geographic origin (available for wild accessions) and large phenotypic variation for fruit quality traits were represented. The 96 mixed core collection will help in understanding domestication of tomato from *S. pimpinellifolium*. Identified alleles of interest in admixed *S. l. cerasiforme *could be assigned to *S. pimpinellifolium *or to *S. lycopersicum *to identify their wild or cultivated origins. Core collections will be used to detect genes associated with domestication i.e. under differential selective constraints in domesticated and wild clusters, and to test their potential for breeding [48]. The 8 and the 32 *S. l. cerasiforme *core collection are interesting for rapid sequencing and identifying SNPs and for evolutionary genomics studies, respectively. These core collections will be of interest for new high-throughput analysis of fruit quality integrating 'omic' information such as metabolomic, proteomic or transcriptomic analysis.

## Conclusion

This study highlighted the unknown genetic structure of our wild and cultivated germplasm, enhancing the understanding of the history of the tomato complex. It clarified the position of *S. l. cerasiforme *in the evolution of the cultivated tomato. Part of this sub-species is genetically close to the cultivated *S. lycopersicum *group and the other part is in admixture between cultivated and wild related groups. This admixed cluster is of high interest for increasing resolution of association genetics. We created nested core collections implemented with accessions from *S. lycopersicum *and *S. pimpinellifolium *that maximize genetic diversity. These core collections are available for the tomato community and can be used as standardized panels for identifying novel interesting genes or polymorphism. Future studies will focus on the characterization of *S. l. cerasiforme *to understand the domestication process in more detail and to prospect for new interesting alleles.

## Methods

### Plant Material

The French collection of wild and cultivated tomato maintained in Institut National de Recherche Agronomique in Avignon (South of France) was used for genotyping. In this collection, most tomato accessions are inbred lines maintained by selfing and characterized for vegetative and reproductive traits. The whole collection consists of nearly 2000 accessions containing inbred cultivars, landraces, and representatives of wild related species. It collates accessions from French researchers' prospecting, from breeders' collections, from the Tomato Genetics Resource Center (Davis, California USA), the Centre for Genetic Resources (Wageningen, Netherlands), the North Central Regional Plant Introduction Station (USA) and from the N.I. Vavilov Research Institute of Plant Industry (St Petersburg, Russia). We used a subset of 360 accessions (see Additional file 3: Individuals information and SSR genotypes) with a majority of *S. lycopersicum *(130 accessions), *S. l*. *cerasiforme *(144 accessions) and *S. pimpinellifolium *(66 accessions). For the red-fruited accessions, classification in different species was based essentially on fruit size [49]. We added one *S. cheesmaniae *and one *S. galapagense *(formerly *L. cheesmanii f. minor*) which are part of red-fruited tomatoes but not included in the studied sample for domestication, because they are assumed to have evolved separately and to be endemic in the Galapagos Islands. Eighteen representatives of wild and green-fruited related were represented by *S. neorickii *(1), *S. chmielewskii *(2), *S. peruvianum *(2), *S. chilense *(2), *S. pennellii *(2) and *S. habrochaites *(2). All red-fruited accessions underwent from one to three cycles of self-pollination. Because of the inbred nature of these accessions, only one plant per accession was used for genotyping. All accessions are available on request from the corresponding author.

*S. l. cerasiforme *accessions (144 accessions) with 39 accessions of *S. lycopersicum *and 19 accessions of *S. pimpinellifolium *were grown in Avignon (South of France) and were phenotyped for growth habit (determinate: *sp *or indeterminate: *sp*^+^), flower stigma insertion (+) or exertion (-), petal length, petal number, green shoulder (presence/absence), stem hairiness (presence/absence), fruit locule number, fruit weight (FW), color in L*a*b* color space: one measure for lightness (L), one measure for the position between red and green (a) and one measure for the position between yellow and blue (b) with a Konica Minolta CR-300 chromameter, firmness with a Durofel durometer , soluble solid content (SSC) and titratable acidity (TA). Phenotypic data were only used for core collection sampling. Quantitative data were split into 5 classes of equi-spaced breaks with class size calculated as [max(X)-min(X)]/5 with X the quantitative variable.

### DNA extraction and Microsatellite genotyping

DNA was isolated from 100 mg frozen leaves using to the DNeasy Plant Mini Kit (Qiagen, Valencia, California, USA). Twenty microsatellite loci were used for genotyping (Table 1). These SSR markers were selected from Sol Genomics Network webpage at .

Amplification reactions were performed according to Ronfort et al. [25]. Samples were prepared by adding 3 μL of diluted PCR product to 6.875 μL formamide and 0.125 μL Gensize 400 HD Rox Size Standard (Applied Biosystems, Foster City, USA). Amplified products were detected on an ABI 3710 × l (Applied Biosystems, Foster City, USA) capillary sequencer. Analyses were performed using the GeneMapper 3.7 sofware (Applied Biosystems, Foster City, USA).

### Diversity analysis

For each microsatellite locus, the number of alleles (N_A_), allelic frequency, the expected (He) and observed (H_o_) heterozygosities were estimated considering both the whole collection and the red-fruited accessions using Genetix 4.05.2 software [50]. Heterozygosity was also compared between subsets at the species level.

### Inference of population structure

To infer the population structure of the tomato collection, we used a model-based clustering algorithm implemented in the computer program Structure version 2.0 (Pritchard, Stephens, and Donnelly, 2000). This algorithm uses a multilocus genotype to identify a predetermined number (K) of clusters that have distinct allele frequencies and assigns portions of individual genomes to these clusters. It proceeds by assuming that observations are randomly drawn from a parametric model and inference for the parameters allows estimation of ancestry probability from each putative cluster, for all individuals. Only *S. lycopersicum*, *S. l. cerasiforme *and *S. pimpinellifolium *accessions were included in this analysis. Since tomato accessions used are highly homozygous (autogamy plus self-pollination of accessions), we used a haploid setting [25,51]. Given the hybrid hypothesis for the *S. l. cerasiforme *variety we used the admixture model assuming correlation among allele frequencies. Ten runs were taken into account for each K value (K is the number of clusters to be inferred), for K ranging from 1 to 15. For each run, we used a burn-period of 500,000 Markov Chain Monte Carlo iterations and then 250,000 iterations for estimating the parameters. Pr(X|K) (i.e. the posterior probability of the data (X) given K) and the associated standard deviation was computed for each simulation and K_opt _was inferred from the formula established by Evanno et al. (2005); K_opt _being the mode of the first peak of ΔK = |L"(K)|/s[Pr(x|k)], with |L"(K)| the absolute value of the second order rate of change of Pr(X|K) with respect to K; and s[Pr(x|k)] the variance of the posterior probability of the data given K. To avoid genetic classification at the species level, Structure2.0 runs were also performed with the same parameters on sub-groups defined by the software but for K ranging from one to ten. For each K_opt_, individuals were assigned into a cluster according to their proportion of membership into this group. Graphical representation of the individual assignation into groups was performed with distruct1.1 software [52]. Analysis of locus by locus MOlecular VAriance (AMOVA) was performed (1000 permutations) and F_ST_, the correlation of alleles within subpopulatons, was calculated (1000 permutations) with Arlequin3.11 [53]. Pairwise comparisons of linkage disequilibrium (LD) among loci were computed with the dedicated procedure of the TASSEL software, using 1,000 permutations.

### Graphical diversity analysis

Genetic uniqueness of each accession was determined with pairwise comparison of multilocus DNA profiles. When two or more accessions had the same profile, only one was taken into account in subsequent analyses. Dissimilarity matrices were built according to the simple matching coefficient [54,55]:

$$d_{ij}=1-\frac{1}{L}\sum_{l=1}^{L}\frac{m_{l}}{\pi }$$

where *L *is the locus number, *π *is the ploidy level and *m *the number of common alleles between individuals *i *and *j*. Bootstraping was performed using 500 replicates for each dissimilarity matrix. Principal coordinate analysis (PCoA) offered graphical representation of genetic distance between accessions and was performed using Darwin 5.0 software [56].

### Core collection sampling

For sampling core collections, we used the Maximization (M) algorithm implemented in MSTRAT software version 4.1 [57], and compared the result to a random strategy. The minimum number of accessions in the core collection to capture all alleles present in the whole sample was evaluated by sampling simulations of this collection. The core collections were built using all SSR data and phenotypic data from 12 morphological traits: growth habit, flower stigma insertion or exertion, petal length, petal number, green shoulder, hairiness, fruit locule number, fruit weight, color in L*a*b* color space and firmness. Soluble Solid Content and Titratable Acidity were used only to validate capture of phenotypic diversity. For evaluation of core collection's minimal size and for individual sampling of the collections, 15 replicates of 30 iterations for each replicate were performed.

## Authors' contributions

NR participated in the conception of the study, analyzed the data and wrote the manuscript. SS participated in the design of the study and was responsible, with NR, for obtaining the molecular data. SM critically revised the manuscript for intellectual content. MC participated in the conception and the coordination of the study and helped to draft the manuscript. All authors read and approved the final manuscript.

## Supplementary Material

Additional file 1 **Determination of Kopt for each species**. This file provides the graphical determination (Evanno, 2005) of Kopt for each species. Click here for file

Additional file 2 **Cerasiforme and mixed core collections**. This file provides a list of the different core collections for *S. lycopersicum *var. *cerasiforme *implemented with *S. lycopersicum *and *S. pimpinellifolium*. Click here for file

Additional file 3 **Individuals information and SSR genotypes (n = 360)**. This file provides a list of all individual genotypic data for 20 SSR markers. Click here for file
