# Supplementary material for: A clarified position for solanum lycopersicum var. cerasiforme in the evolutionary history of tomatoes (solanaceae)
Source: BMC Plant Biol. 2008 Dec 20;8:130. doi: 10.1186/1471-2229-8-130 (PMC2657798; doi:10.1186/1471-2229-8-130)
Supplement: Additional file 1 — Determination of Kopt for each species. This file provides the graphical determination (Evanno, 2005) of Kopt for each species. [file 1471-2229-8-130-S1.ppt]

## Slide 1
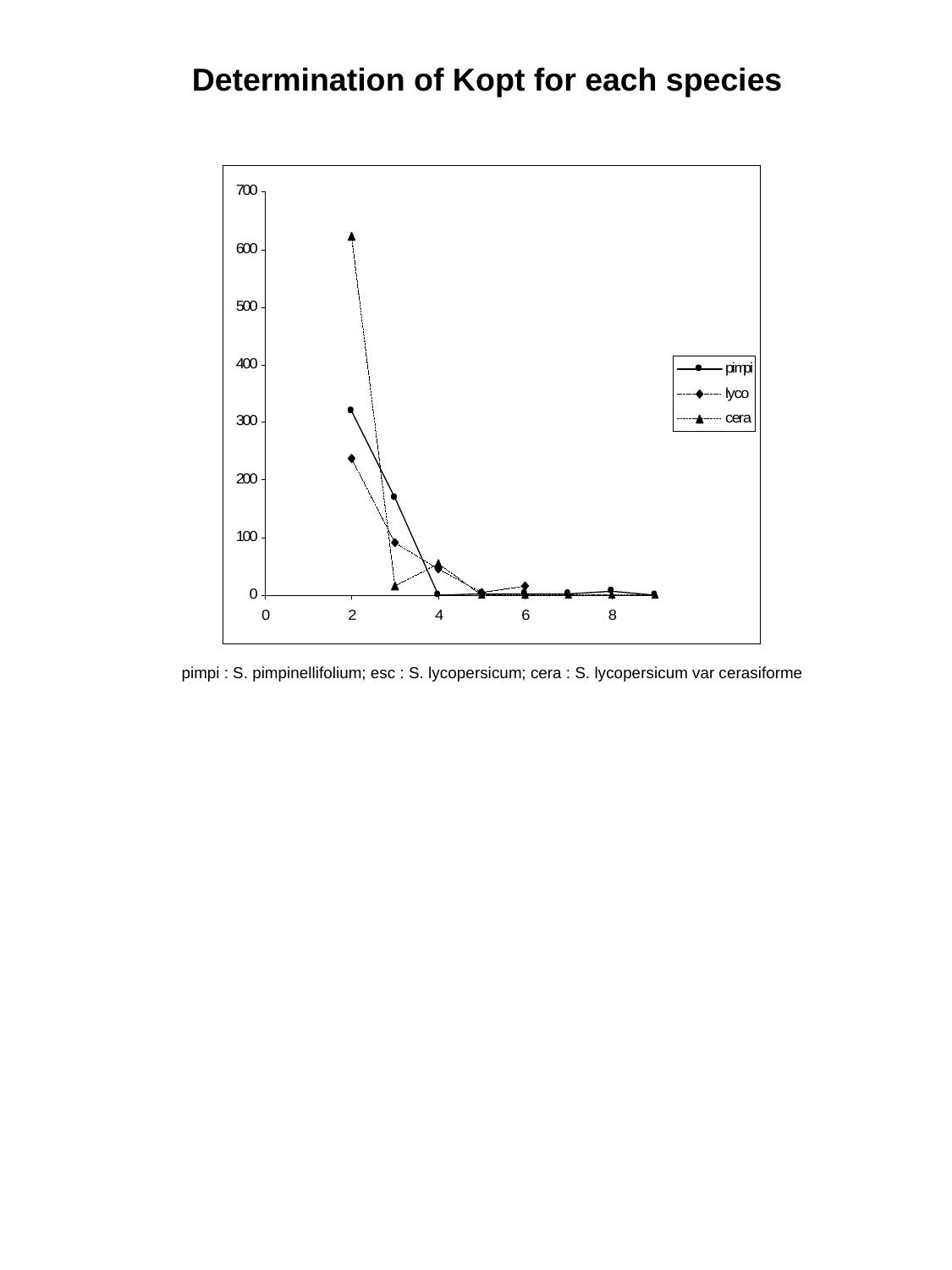

Determination of Kopt for each species
pimpi : S. pimpinellifolium; esc : S. lycopersicum; cera : S. lycopersicum var cerasiforme
